# Pericarditis

**Published:** 1848-06

**Authors:** 


*Pericarditis.*—It is important to notice that the pains accompanying chest diseases are often referred by the patient to situations inferior, in point of position, to those organs actually affected. This accounts for the frequency of liver affection as a clinical disease, and the rarity of its detection on post-mortem examination. The pains of pericarditis are mostly referred to the scrobiculus cordis or hypochondrium. In one case where Jaksch, misled by these symptoms, diagnosed inflammation of the diaphragm, pericarditic effusion was found after death.—*Clinical Notes taken in the Hospital of Prague.*

